# Wild carrot pentane-based fractions suppress proliferation of human HaCaT keratinocytes and protect against chemically-induced skin cancer

**DOI:** 10.1186/s12906-016-1531-0

**Published:** 2017-01-10

**Authors:** Wassim N Shebaby, Mohamad A Mroueh, Petra Boukamp, Robin I Taleb RI, Kikki Bodman-Smith, Mirvat El-Sibai, Costantine F Daher

**Affiliations:** 1 Department of Natural Sciences, School of Arts and Sciences, Lebanese American University, P.O. Box 36, Byblos, Lebanon; 2 School of Pharmacy, Department of Pharmaceutical Sciences, Lebanese American University, P.O. Box 36, Byblos, Lebanon; 3 Deutsches Krebsforschungszentrum DKFZ, German Cancer Research Center, Genetics of Skin Carcinogenesis, Im Neuenheimer Feld 280, Heidelberg, 69120 Germany; 4 IUF–Leibniz Research Institute for Environmental Medicine, Duesseldorf, Germany; 5 Faculty of Health and Medical Sciences, Department of Microbial and Cellular Sciences, University of Surrey, Guildford, UK

**Keywords:** *Daucus carota*, Wild carrot, HaCaT, TPA/DMBA skin cancer

## Abstract

**Background:**

Previous studies in our laboratory showed that the Lebanese *Daucus carota* ssp. carota (wild carrot) oil extract possesses in vitro and in vivo anticancer activities. The present study aims to examine the cytotoxic effect of *Daucus carota* oil fractions on human epidermal keratinocytes and evaluate the chemopreventive activity of the pentane diethyl ether fraction on DMBA/TPA induced skin carcinogenesis in mice.

**Methods:**

Wild carrot oil extract was chromatographed to yield four fractions (F1, 100% pentane; F2, 50:50 pentane:diethyl ether; F3, 100% diethyl ether; F4 93:7 chloroform:methanol). The cytotoxic effect of fractions (10, 25, 50 and 100 μg/mL) was tested on human epidermal keratinocytes (non-tumorigenic HaCaT cells and tumorigenic HaCaT-ras variants) using WST a ssay. Cell cycle phase distribution of tumorigenic HaCaT-ras variants was determined by flow cytometry post-treatment with F2 fraction. Apoptosis related proteins were also assessed using western blot. The antitumor activity of F2 fraction was also evaluated using a DMBA/TPA induced skin carcinoma in Balb/c mice.

**Results:**

All fractions exhibited significant cytotoxicity, with HaCaT cells being 2.4–3 times less sensitive than HaCaT-ras A5 (benign tumorigenic), and HaCaT-ras II4 (malignant) cells. GC-MS analysis revealed the presence of a major compound (around 60%) in the pentane/diethylether fraction (F2), identified as 2-himachalen-6-ol. Treatment of HaCaT-ras A5 and HaCaT-ras II4 cells with F2 fraction resulted in the accumulation of cells in the sub-G1 apoptotic phase and decreased the population of cells in the S and G2/M phases. Additionally, F2 fraction treatment caused an up-regulation of the expression of pro-apoptotic (Bax) and down-regulation of the expression of anti-apoptotic (Bcl2) proteins. A decrease in the phosphorylation of AKT and ERK was also observed. Intraperitoneal treatment with F2 fraction (50 or 200 mg/kg) in the DMBA/TPA skin carcinogenesis mouse model showed a significant inhibition of papilloma incidence (mice with papilloma), yield (number of papilloma/mouse) and volume (tumor relative size) at weeks 15, 18 and 21.

**Conclusion:**

The present data reveal that F2 fraction has a remarkable antitumor activity against DMBA/TPA-induced skin carcinogenesis, an effect that may be mediated through inhibition of the MAPK/ERK and PI3K/AKT pathways.

**Electronic supplementary material:**

The online version of this article (doi:10.1186/s12906-016-1531-0) contains supplementary material, which is available to authorized users.

## Background

Skin cancer is one of the most common types of cancer accounting for at least 40% of cases worldwide and is even more common among Caucasians [1]. Carcinogenesis is a multistage process and it is characterized by three stages including initiation, promotion and progression. Synthetic or natural agents, which may hinder, halt or reverse the process of carcinogenesis at different stages, would be considered an effective approach in cancer prevention and treatment. Medicinal plants have been reported to inhibit tumor formation in vivo by blocking the initiation phase or slowing down the promotion and progression phases of carcinogenesis [2–4]. Conventional chemotherapy or radiotherapy are often accompanied with adverse side-effects. Therefore, there is an increasing interest in pharmacological evaluation of various natural products that possess anticancer and chemopreventive properties with limited side effects [5].

*Daucus carota* (Linnaeus) ssp. carota, known as wild carrot, is a member of the family Umbelliferae (Apiacae). The plant grows in moderate regions throughout the world [6] and is commonly consumed as a salad in the Mediterranean diet or used as an additive in some recipes [7]. In Lebanon, it is traditionally used for the treatment of gastric ulcer, diabetes, muscle pain and cancer. The plant has been also reported to exhibit antilithic, diuretic [6, 8] antibacterial, and antifungal activities [9, 10]. Previous studies in our laboratory revealed that *Daucus carota* oil extract (DCOE) possesses antioxidant [11], anti-inflammatory and anti-ulcer activities [12]. It also exhibited potent cytotoxicity against colon (Caco-2, HT-29), breast (MCF-7, MDA-MB-23) [11] and human acute myeloid leukemia cells [13], as well as significant anti-tumor promoting effect against DMBA/TPA skin carcinogenesis in mice [4]. Recently, DCOE was subjected to chromatographic separation and fractions were shown to possess antioxidant, hepatoprotective [14] as well as anticancer activity in vitro [14, 15]. The pentane/diethyl ether fraction (F2) containing a major compound identified as 2-himachalen-6-ol (61.4%), was found to suppress proliferation of HT-29 cells by inducing apoptosis [14]. It also inhibited motility of MDA-MB231 and SF-268 cells and reduced invasion of B16F-10 cells [15].

Keratinocytes are the most abundant cell type making more than 95% of the skin cells. There are three main types of skin cancer: melanoma, Basal cell carcinoma (BCC) and squamous cell carcinoma (SCC). The most common types of cancer in humans are the ones that directly target keratinocytes and include basal cell carcinomas (BCC) and squamous cell carcinomas (SCC) [16]. In contrast, melanoma is the least common, but the most aggressive type of skin cancer and it is responsible for the majority of skin cancer mortalities. Previous work in our laboratory revealed that DCOE exhibited remarkable antitumor-promoting activity against DMBA/TPA induced squamous cell carcinoma (papilloma) in mice. The first aim of this study, therefore, was to evaluate the in vitro anti-proliferative effect of F2 fraction in human skin carcinogenesis HaCaT model with its three different stages of tumorigenicity (non-tumorigenic, non-invasive and invasive). These cells are considered the counterparts of the chemically induced squamous cell carcinoma in the DMBA/TPA skin carcinogenesis model in mice. The second aim of this study was to further assess the in vivo antitumor efficacy of F2 fraction in DMBA/TPA skin carcinogenesis mouse model.

## Methods

### Chemicals and reagents

Dimethyl sulphoxide (DMSO), Dulbecco’s modified Eagle’s medium (DMEM), Trypsin, 7,12-Dimethylbenz (a) anthracene (DMBA), 12-O-tetradecanoylphorbol-13-acetate (TPA), were purchased from Sigma-Aldrich, St. Louis, USA. WST-1 reagent was purchased from Roche, Mannheim, Germany. Silica gel 60 was purchased from ACROS organics, New Jersey, USA and Silica gel 40 (35–70) mesh were purchased from Sigma Aldrich, St. Louis, USA. All other chemicals used in the experiments were of analytical grade.

### Sample collection and oil extraction

*Daucus carota* (Linnaeus) ssp. carota mature umbels (closed, yellow-brownish) were collected at the post flowering season between July and August from Byblos, Lebanon. The plant was identified according to the characteristics described in the “Handbook of Medicinal Herbs” [6] and confirmed by Dr. A. Houri, a Lebanese plant expert at the Lebanese American University. A voucher specimen of the plant material used in this study has been deposited in the Department of Natural Sciences herbarium. The extraction procedure was carried out according to the method described by Zeinab et al. [4]. Briefly, *Daucus carota* umbels were air dried in the shade and then cut into small pieces for oil extraction in methanol/acetone (1:1) for 72 h. The extract was then filtered and evaporated to dryness under reduced pressure. The residue was centrifuged and the oil was dried over anhydrous sodium sulfate. The final yield (3.47%) was stored in a closed amber bottle at 4 °C until use.

### DCOE fractionation

DCOE fractionation was carried out as described previously [14]. Briefly, thirty grams of DCOE were chromatographed on a silica gel column (size 35–70 mesh), eluted with pentane (100%), pentane: diethyl ether (50:50), diethyl ether (100%) and chloroform: methanol (93:7) to yield F1, F2, F3 and F4 fractions respectively. Fractions were analyzed by TLC using hexane:ethyl acetate (70:30) as mobile phase and plates were stained with 2% anisaldehyde. The chemical components of various fractions were determined as previously described [17].

### Animals

All in vivo experiments were performed on male adult BALB/c mice (6 weeks old) bred in the animal facility of the Biology Department at the Lebanese American University. Mice, weighing 18–21 g were housed under optimum conditions of temperature set at 22 ± 2 °C, humidity at 50 ± 5% and an alternating cycle of light and dark. The animals were supplied with standard laboratory chow diet and water. All experimental protocols were approved by the Department of Natural Sciences Animal Ethical Committee, which complies with the Guide for the Care and Use of Laboratory Animals (Committee for the Update of the Guide for the Care and Use of Laboratory Animals, 2010).

### Cell lines and culture

The human HaCaT keratinocytes, HaCaT (non-tumorigenic), HaCaT-ras A5 (benign tumorigenic) and HaCaT-ras II4 (malignant) [18, 19] were cultured in Dulbecco’s modified Eagle’s medium (DMEM) supplemented with 10% heat-inactivated fetal bovine serum (FBS), 100 μg/mL streptomycin and 100 U/mL penicillin. Cells were maintained in a humidified atmosphere containing 5% CO_2_ at 37 °C.

### Cell proliferation assay

Proliferation of the HaCaT and HaCaT-ras variants was tested using the WST-1 assay. Cells were plated in 96-welled plates at a concentration of 5×10^4^ cell/mL for 24 h. Afterwards, the three cell lines were treated with increasing concentrations (10, 25, 50 and 100 μg/mL) of DCOE fractions dissolved in DMSO for 48 h. Cell survival was assessed using WST-1 reagent (Roche Applied Science, Penzberg, Germany). The color intensity was quantified at 450 nm using Multiskan FC microplate ELISA reader (Thermo fisher Scientific, Rockford, IL, USA).

### Cell cycle analysis

The effect of F2 fraction on cell cycle distribution was assessed by flow cytometry after staining of HaCaT-ras A5, and HaCaT-ras II4 cells with propidium iodide (PI). Briefly, both cells lines (1x10^5^cells/mL) were treated with different concentrations (25 and 50 μg/mL) of F2 fraction and cultured in 6-well plates for 48 h. The treated cells were harvested, washed with PBS and fixed with 70% ethanol on ice. The cells were then washed with cold PBS, suspended in 200 μL 1× Propidium Iodide + RNase staining solution and incubate at 37 °C in the dark for 30 min. Propidium Iodide Flow Cytometry Kit (Abcam, Cambridge, UK) was used for cell cycle analysis. The DNA content of the cells was measured by C6 flow cytometer (BD Accuri Cytometers, Ann Arbor, MI USA) and the population of each phase was determined using CFlow Plus analysis software (BD Accuri Cytometers, Ann Arbor, USA).

### Western blot

HaCaT-ras II4 cells were treated with two different concentrations of F2 (25 and 50 μg/mL) for 48 h. Adherent and non-adherent cells were collected on ice, washed twice with PBS, lysed with lysis buffer and centrifuged at 12,000 g for 10 min at 4 °C. The cell lysate was heated at 100 °C for 5 min, and the protein content was determined by the Bio-Rad protein assay (Bio-Rad, Hercules, CA, USA).

Equal protein concentrations were subjected to Western blot analysis as described previously [20]. The PVDF membranes were blocked with blocking buffer (1× TBS, 0.1% Tween-20, 5% skim milk) for 2 h and then probed with primary rabbit polyclonal antibodies to Caspase-3, AKT, p-AKT, ERK and p-ERK (Abcam, Cambridge, UK) and mouse monoclonal antibodies to actin, p53, p21, Bcl-2, BAX (Santa Cruz, CA) at dilution ranging from 1/1000 – 1/5000 at 4 °C overnight. Later, the primary antibodies were washed away with TBST for 2 h and the membranes were treated with horseradish peroxidase (HRP)-coupled secondary antibodies in blocking buffer at a dilution of 1/5000 (Abcam, Cambridge, UK) for 1 h, and washed with TBST afterwards. Finally, detection of each protein was performed using the chemiluminescence ECL kit (Abcam plc, Cambridge, UK). Blot images were obtained with the image lab Software (BioRad, Chemidoc imaging instrument).

### Tumor promotion experiment

The dorsal surface of BALB/c mice was shaved using electric clippers. Papillomas were initiated by a single topical application of 190 nmol of 7,12-Dimethylbenz (a) anthracene (DMBA) in 0.2 mL acetone. Three weeks after initiation, the shaved area of the back of the mice were treated by topically applying 8 nmol TPA in 0.2 mL acetone twice weekly till the end of the experiment [21]. Before TPA promotion (30 min), three groups of 10 mice each received intraperitoneal injections of F2- fraction (10 mg/kg, 50 mg/kg, 200 mg/kg) dissolved in DMSO. Control group (*n* = 10) received an intraperitoneal injections of DMSO. Papilloma development was followed weekly for 21 weeks. Tumor incidence was assessed as the percentage of mice in each group having tumors on their back. The tumor volume was measured at week 15, 17, 20. Tumor volume was estimated in comparison to specific reference volume models. Briefly, papillomas length and width were measured using a caliper and matched with similar clay tumor models of known standardized volumes [16]. Papilloma smaller than 1 mm in diameter were not counted. Mice in each group were weighed at week 5, 13 and 21.

### Statistical analysis

In vitro data were analyzed for statistical significance using one way analysis of variance (ANOVA). Values of the different tested parameters within each group are presented as mean ± SEM. Significant main effect differences were tested using Bonferroni post hoc test for multiple comparisons. All data were analyzed with the statistical package SPSS 18, and differences between groups were considered statistically significant if *p* < 0.05. The IC_50_ was calculated by a nonlinear regression curve with the use of Prism Graph Pad Prism version 5.0 software for Windows. In the in vivo study, statistical analysis was done using Linear Mixed Effect Models in R (3.2.2) with the function lie from the name package. Two models were built, one for the number of papilloma and another for the papilloma volume. In both models, the time and dosage were considered as fixed effects while the mice id was considered as a random effect. Raw data are available in a supporting Excel (Additional file 1) file presented along with the manuscript.

## Results

### Effect of DCOE fractions on cell survival

**Fig. 1:**
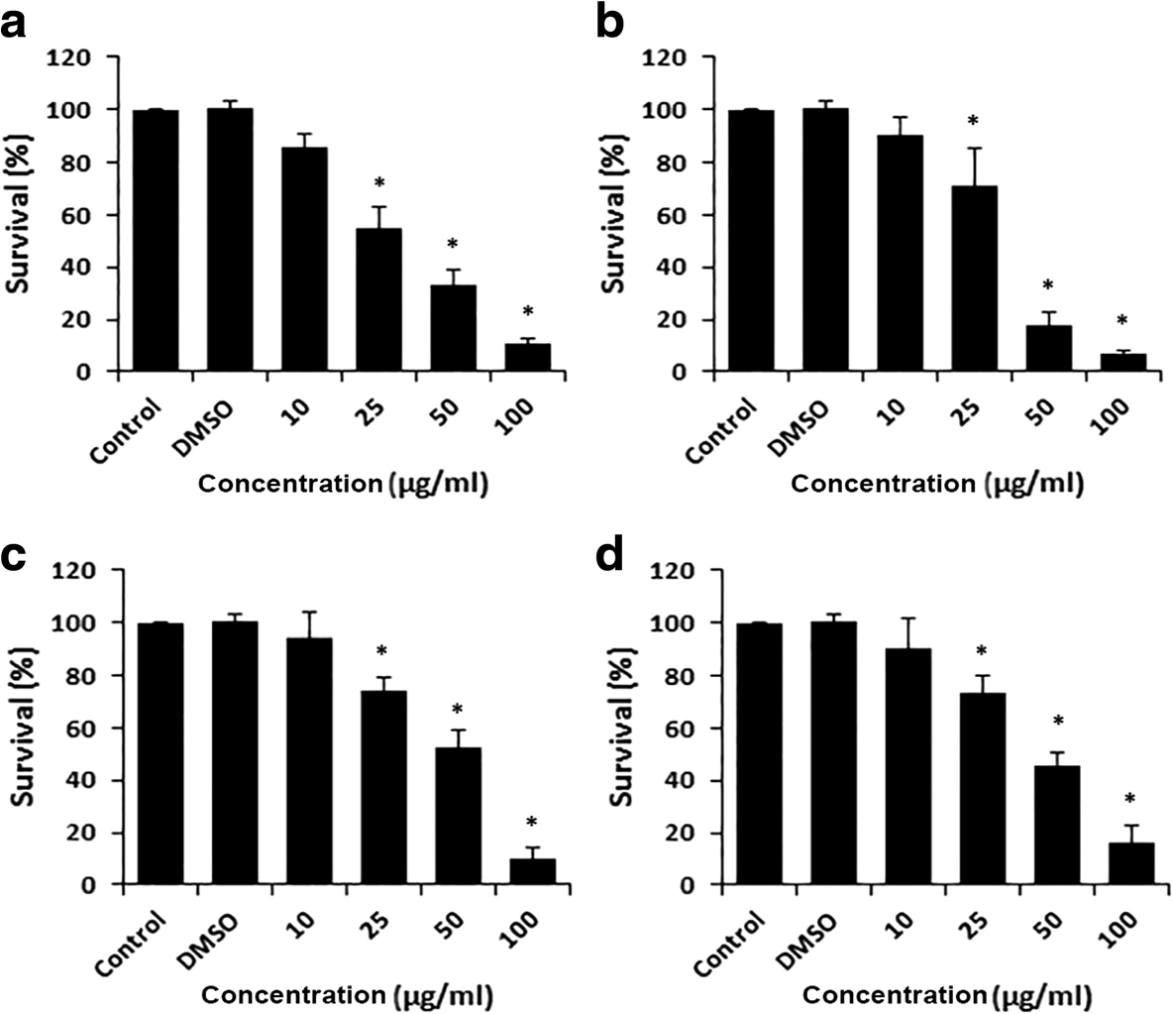
Antiproliferative effect of DCOE fractions on HaCaTcells (non-tumorigenic). Cells were treated with F1-fraction (**a**), F2-fraction (**b**), F3-fraction (**c**), F4-fraction (**d**) at different concentrations (10, 25, 50 and 100 μg/mL) or with 0.5% DMSO for 48 h followed by measurement of cell proliferation by WST-1 assay. Data are expressed as % survival of cells. Data are the mean ± SEM from three independent experiments. * denotes *P* < 0.05 vs. DMSO group as measured by one-way ANOVA

**Fig. 2:**
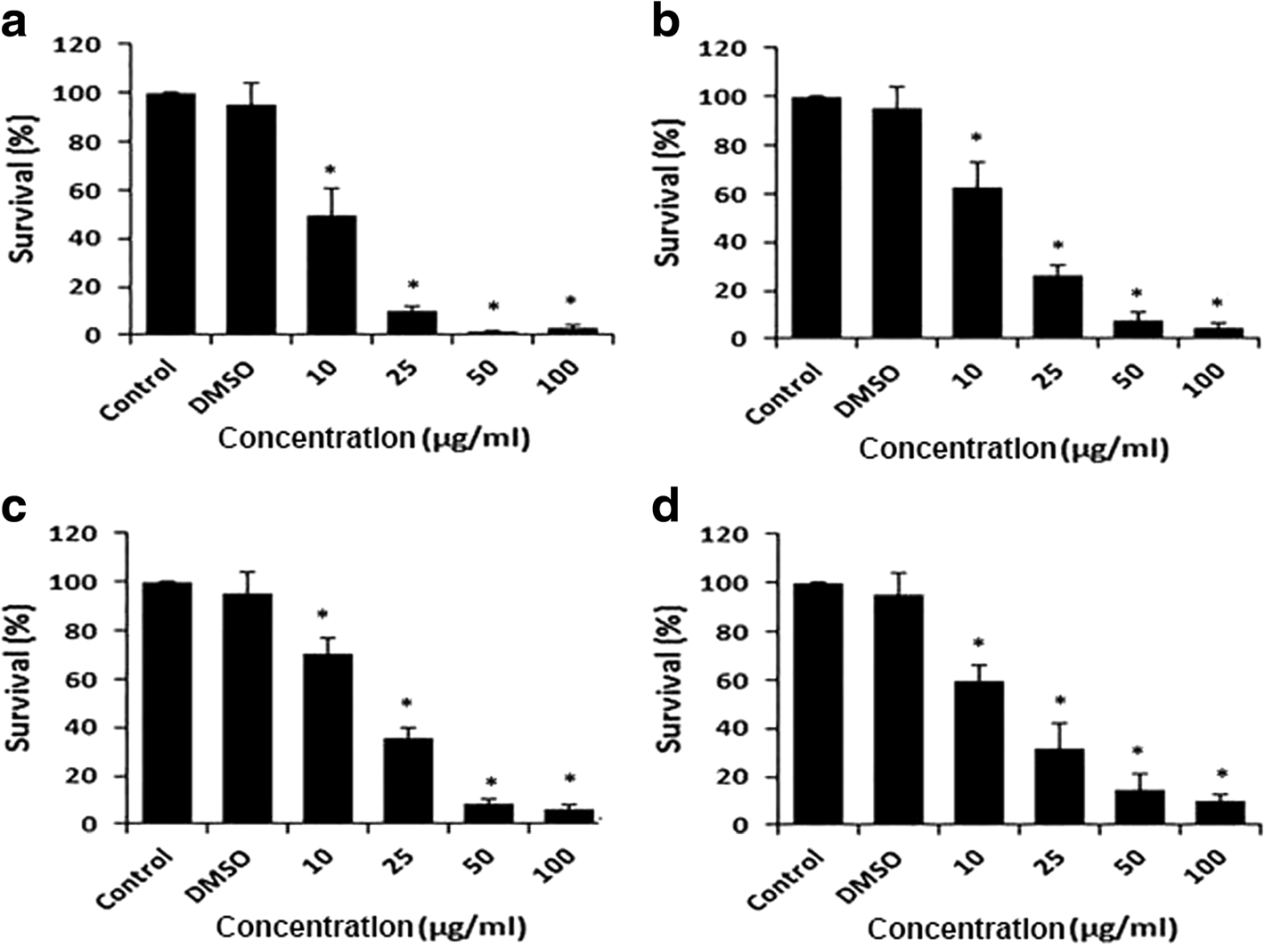
Antiproliferative effect of DCOE fractions on the HaCaT-ras A5 cells (benign tumorigenic). Cells were treated with F1-fraction (**a**), F2-fraction (**b**), F3-fraction (**c**), F4-fraction (**d**) at different concentrations (10, 25, 50 and 100 μg/mL) or with 0.5% DMSO for 48 h followed by measurement of cell proliferation by WST-1 assay. Data are expressed as % survival of cells. Data are the mean ± SEM from three independent experiments. * denotes *P* < 0.05 vs. DMSO group as measured by one-way ANOVA

**Fig. 3:**
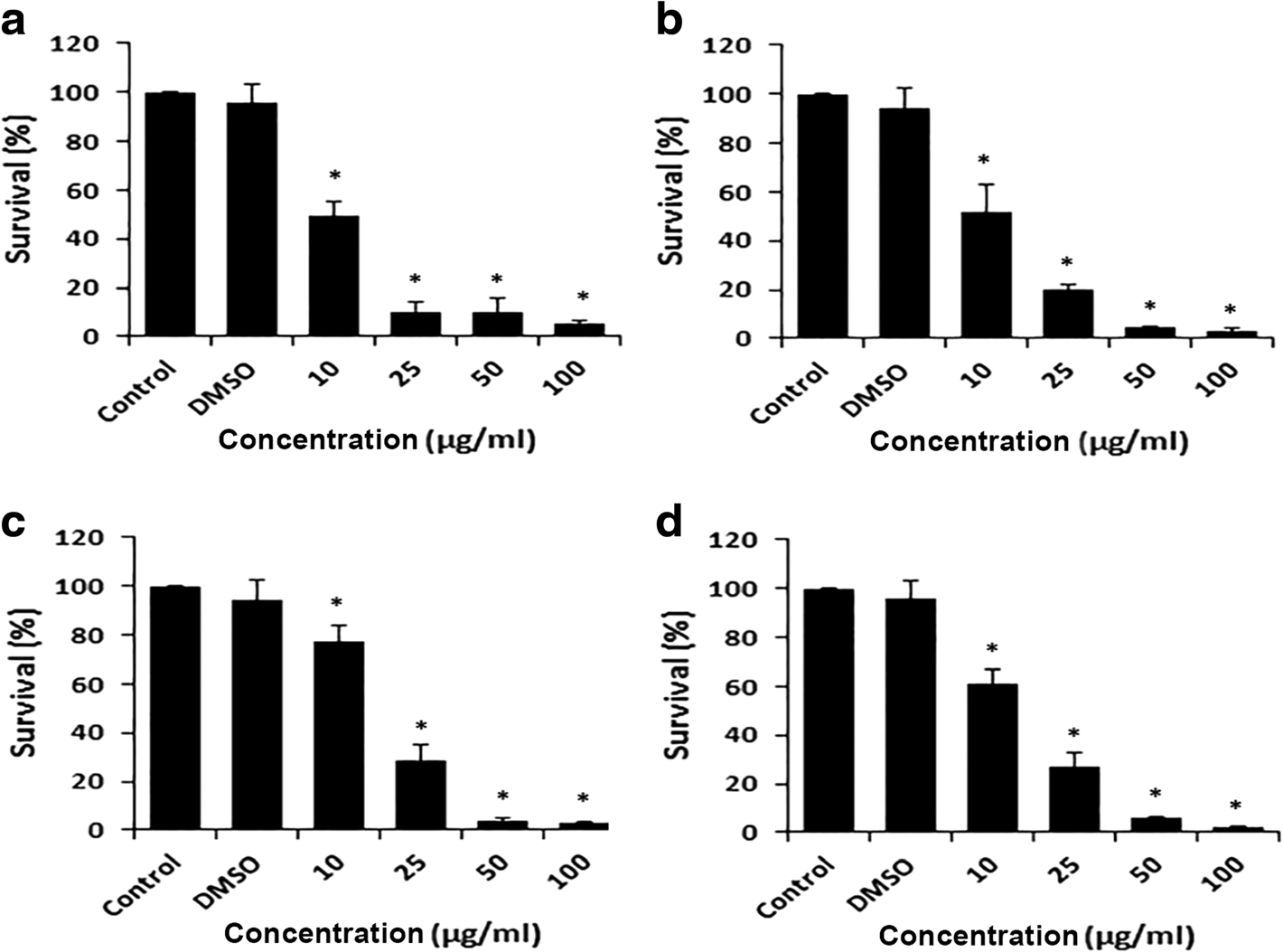
Antiproliferative effect of DCOE fractions on the HaCaT-ras II4 cells (malignant). Cells were treated with F1-fraction (**a**), F2-fraction (**b**), F3-fraction (**c**), F4-fraction (**d**) at different concentrations (10, 25, 50 and 100 μg/mL) or with 0.5% DMSO for 48 h followed by measurement of cell proliferation by WST-1 assay. Data are expressed as % survival of cells. Data are the mean ± SEM from three independent experiments. * denotes *P* < 0.05 vs. DMSO group as measured by one-way ANOVA

The effects of the four fractions on cell survival were examined 48 h post-treatment. The data presented in Figs. 1, 2 and 3 show that all three HaCaT variants exhibited a dose-dependent decrease in cell survival. F1 and F2 demonstrated the strongest (*p* < 0.05) inhibitory effect when compared with F3 and F4 fractions. Data also revealed that the non-tumorigenic HaCaT cells were almost 2.4–3 fold more resistant to cytotoxicity than the two malignant variants (*p* < 0.05). The IC_50_ values of F1, F2, F3 and F4 fractions for all three cell lines are presented in Table 1. In all tested cells, DMSO did not show any significant effect on viability.

**Table 1:** The IC50 (μg/ml) of DCOE fractions against different human skin tumorigenic and non-tumorigenic cell lines

Fractions	Cell line		
	HaCaT-ras A5	HaCaT-ras II4	HaCaT
F1	10.6 ± 0.58^a,1^	10.2 ± 0.90^a,1^	29.8 ± 0.58^a,2^
F2	14.6 ± 0.76^b,2^	11.4 ± 0.67^a,1^	33.2 ± 0.60^b,3^
F3	19.1 ± 0.98^d,1^	17.8 ± 0.62^c,1^	47.3 ± 0.75^d,2^
F4	16.2 ± 0.96^c,1^	14.5 ± 0.58^b,1^	43.4 ± 1.11^c,2^

Values are the mean ± SEM from three experiments. Values in a column not sharing a common superscript letter are significantly different (*p* < 0.05) and values in a row not sharing a common superscript number are significantly different (*p* < 0.05)

### Effect of F2 fraction on cell cycle

**Fig. 4:**
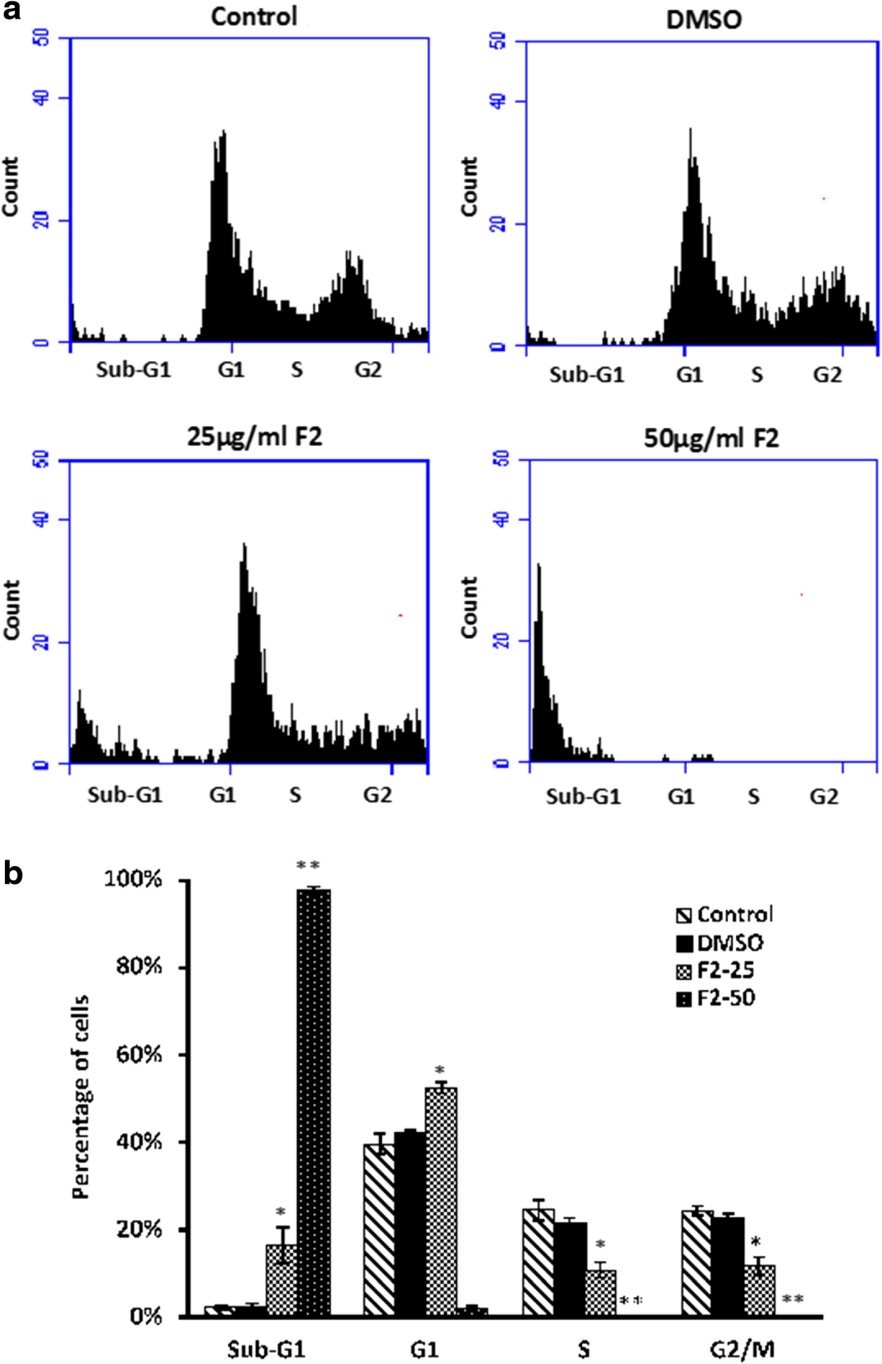
Effect of the F2 fraction on the cell cycle distribution of the HaCaT-rasII4 cells. **a** Cells were treated with either 25 or 50 μg/ml of F2 fraction or with 0.5% DMSO for 48 h, after which cells were stained with PI and analyzed for DNA content by flow cytometry. The sub-G1 peak is considered as the apoptotic portion. The results shown are representative of three independent experiments. **b** Bar graphs showing the percentages of cell population of each phase of the cell cycle. Data are means ± SEM of three independent experiments. * denotes *P* < 0.05 and ** denotes *P* < 0.01 vs. DMSO group as measured by one-way ANOVA

**Fig. 5:**
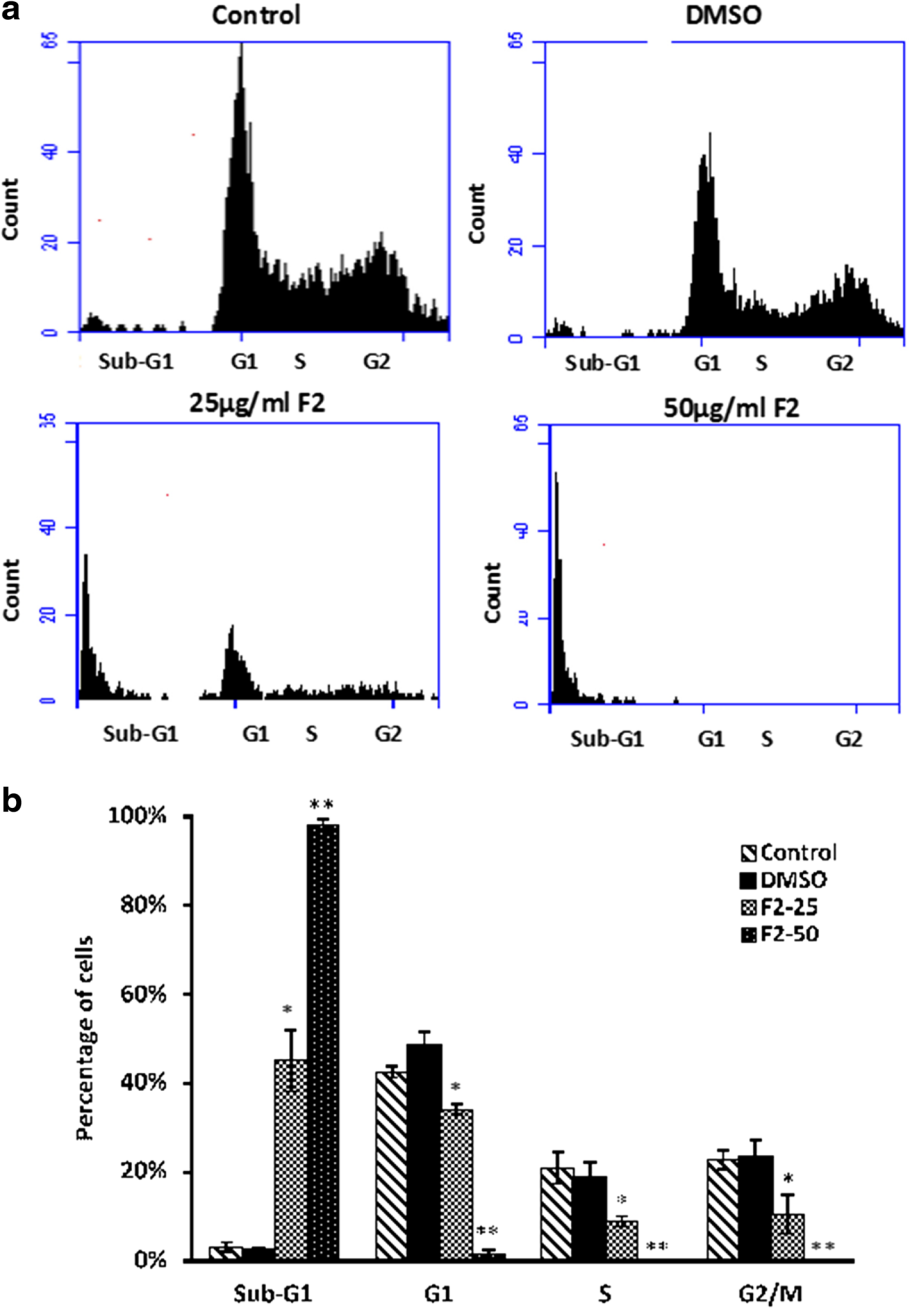
Effect of the F2 fraction on the cell cycle distribution of the HaCaT-rasA5 cells. **a** Cells were treated with either 25 or 50 μg/ml of F2 fraction or with 0.5% DMSO for 48 h, after which cells were stained with PI and analyzed for DNA content by flow cytometry. The sub-G1 peak is considered as the apoptotic portion. The results shown are representative of three independent experiments. **b** Bar graphs showing the percentages of cell population of each phase of the cell cycle. Data are means ± SEM of three independent experiments. * denotes *P* < 0.05 and ** denotes *P* < 0.01 vs. DMSO group as measured by one-way ANOVA

Flow cytometric analysis was carried out to determine the effect of F2 fraction on the cell cycle distribution. Following a 48 h exposure to 25 μg/mL of F2, the fraction of sub-G1 phase increased in HaCaT-ras II4 cells from 2.24% (control) to 16.52% (Fig. 4). Similarly, the fraction of cells in G1 phase increased from 42.15–52.53%. Similarly, the number of HaCaT-ras A5 cells in the sub-G1 phase increased significantly (*p* < 0.01) as compared to DMSO control group (Fig. 5). This increase coincided with considerably fewer cells in the S and G2/M phases (*p* < 0.05 and *p* < 0.01). Additionally, treatment of both cell lines with 50 μg/mL of F2 significantly increased the cell population in the sub-G1 to more than 96% with hypodiploid nuclei, indicating that F2 may have caused DNA fragmentation which is a characteristic of late apoptosis.

### Effect of F2 fraction on the expression of apoptosis related proteins in HaCat-II4 cells

**Fig. 6:**
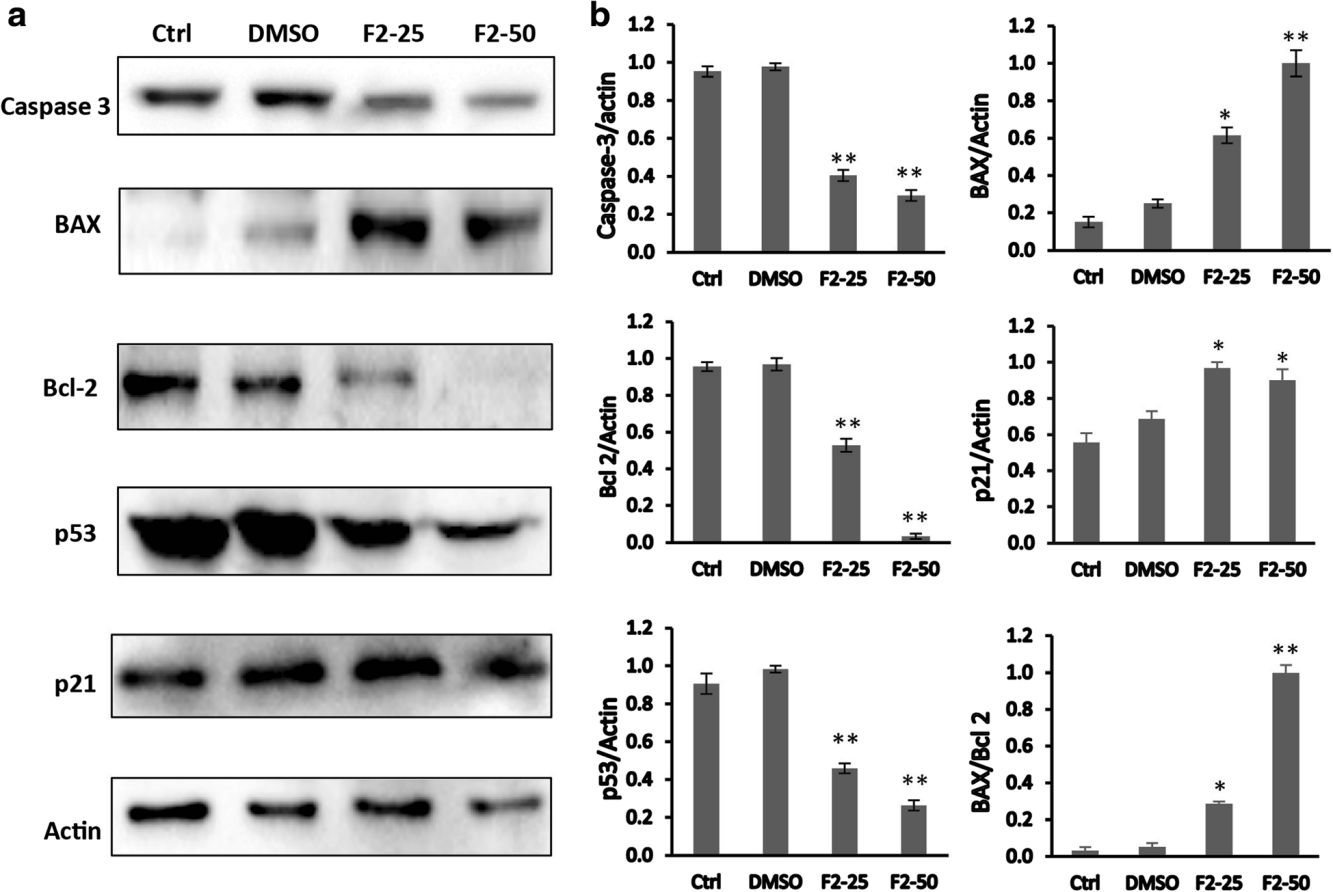
Western blot analysis of apoptosis related proteins in HaCaTras II4 cells. **a** Effect of the F2 fraction on the expression level of p21, p53, caspase-3, Bcl-2 and Bcl-2-associated X (BAX) proteins. Expression of β-actin was used as an internal control. Cells were treated with 25 and 50 μg/ml of F2-fraction or with 0.5% DMSO for 48 h. Western blots are representative of three independent experiments. **b** The densitometer-intensity data of the proteins of each blot is presented as mean ± SEM from three independent experiments. * denotes *P* < 0.05 and ** denotes *P* < 0.01 vs. DMSO as measured by one-way ANOVA

To explore the mechanism by which F2 fraction induces apoptosis, the expression levels of several pro-apoptotic and anti-apoptotic proteins were examined by western blot. Data shown in Fig. 6 revealed that treatment of cells with F2 (25 and 50 μg/mL) caused a significant decrease in the level of caspase-3 (*p* < 0.05). While the level of the anti-apoptotic protein Bcl-2 declined significantly after treatment with the same doses of F2 (*p* < 0.05 and *p* < 0.01 respectively), the level of pro-apoptotic protein BAX was significantly increased (*p* < 0.05 and *p* < 0.01 respectively). Additionally, F2 treatment caused an increased expression of p21 but it decreased that of p53.

### Effect of F2 fraction on PI3K-AKT and MAP kinase pathways in HaCat-II4 cells

**Fig. 7:**
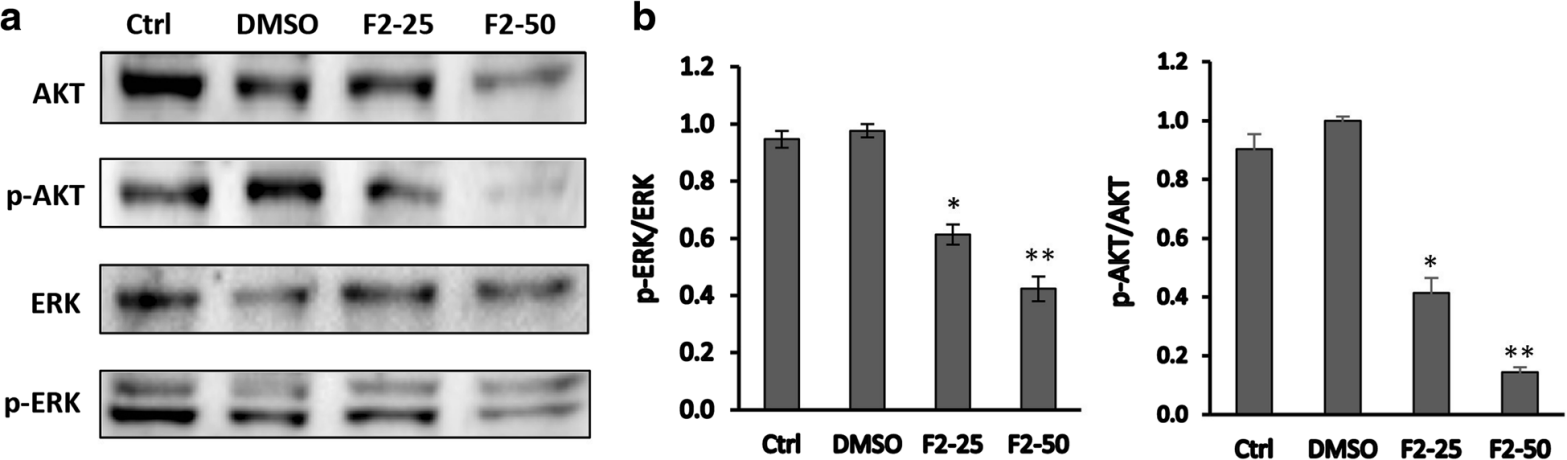
Western blot analysis of the PI3K/Akt and MAPK/ERK? pathways in HaCaT-ras -II4 cells. **a** Effect of the F2 fraction on the expression level of AKT, p-AKT, ERK, p-ERK proteins. Cells were treated with 25 and 50 μg/ml of F2-fraction and cells treated with 0.5% DMSO for 48 h. Western blots were representative of three independent experiments. **b** The densitometer-intensity data of the proteins of each blot is presented as mean ± SEM from three independent experiments. * denotes *P* < 0.05 and ** denotes *P* < 0.01 vs. DMSO as measured by one-way ANOVA

The phosphatidylinositol 3-kinase (PI3K)-AKT and MAP kinase signaling pathways are known to regulate several cellular functions such as proliferation, and apoptosis. Therefore, PI3K activation was measured using Western blot analysis to look for the relative expression of phosphorylated AKT (p-AKT) in relation to non-phosphorylated AKT in HaCaT-ras II4 treated cell lysates. On the other hand, the activity of the MAPK pathway was assessed through examining the level of phosphorylation of ERK. The results revealed that F2 fraction caused a significant dose-dependent decrease in the expression of p-AKT and p-ERK in comparison with the DMSO-treated groups (Fig. 7).

### Effect of the F2 fraction on tumor promotion in mice

The chemopreventive effect of F2 fraction (10, 50 or 200 mg/kg) was monitored for 21 weeks. The percentage papilloma incidence (mice with papillomas) and the average number of papillomas per mouse are available in Additional file 2. Papilloma incidence in the control (DMSO) group as well as in the treated groups was recorded as early as week 6 and increased rapidly to reach 100% at week 10 for the DMSO group. In all treated groups, papilloma incidence reached 90% at week 13. Tables 2, 3 and 4 show that the percentage of papilloma incidence in the control group remained 100% at weeks 15, 18 and 21, whereas in the F2-treated groups (10, 50 and 200 mg/kg) papilloma incidence significantly decreased. Inhibition of papilloma incidence was maximal at 200 mg/kg with 50% by the end of the experiment at week 21 (Table 4).

**Table 2:** The inhibitory effects of different concentrations of F2-fraction on the promotion of mouse skin papilloma at week 15 in tumor-bearing mice

			Papilloma	Yield	Papilloma	Incidence
Treatments	% Survival	Weeks of 1^st^ PAPS	PAPS/Mouse	% Inhibition	% Mice with PAPS	% Inhibition
Control (DMSO)	100	6	14.7 ± 0.55	0	100	0
10 mg/Kg	100	6	12.5 ± 0.34^*^	15	90	10
50 mg/Kg	100	7	12.2 ± 0.33^*^	17	80	20
200 mg/Kg	100	7	9.1 ± 0.46^*^	38	80	20

Values are mean ± SEM. n = 10

^*^ *P* < 0.05

**Table 3:** The inhibitory effects of different concentrations of F2-fraction on the promotion of mouse skin papillomaat week 18 in tumor-bearing mice.

			Papilloma	Yield	Papilloma	Incidence
Treatments	% Survival	Weeks of 1^st^ PAPS	PAPS/Mouse	% Inhibition	% Mice with PAPS	% Inhibition
Control (DMSO)	100	6	13.6 ± 0.30	0	100	0
10 mg/Kg	100	6	11.9 ± 0.32^*^	13	80	20
50 mg/Kg	100	7	10.9 ± 0.35^**^	20	60	40
200 mg/Kg	100	7	8.8 ± 0.44^**^	35	60	40

Values are mean ± SEM. n = 10

^*^ *P* < 0.05, ^**^ *P* < 0.001 vs control

**Table 4:** The inhibitory effects of different concentrations of F2-fraction on the promotion of mouse skin papillomaat week 21 in tumor-bearing mice.

			Papilloma	Yield	Papilloma	Incidence
Treatments	% Survival	Weeks of 1^st^ PAPS	PAPS/Mouse	% Inhibition	% Mice with PAPS	% Inhibition
Control (DMSO)	100	6	13.3 ± 0.29	0	100	0
10 mg/Kg	100	6	10.9 ± 0.52^*^	18	80	20
50 mg/Kg	100	7	9.6 ± 0.50^**^	28	60	40
200 mg/Kg	100	7	7.7 ± 0.71^**^	42	50	50

Values are mean ± SEM. n = 10

^*^ *P* < 0.05, ^**^ *P* < 0.001 vs control

Treatment with F2 (10, 50 and 200 mg/kg) not only inhibited papilloma incidence but decreased also the number of papillomas per mouse in a dose-dependent manner (Additional file 2). At week 15, F2 inhibited papilloma yield by 15, 17 and 38%, respectively (Table 2). At week 18, the inhibition was 13, 20 and 35% (Table 3), and at week 21 it reached 18, 28 and 42%, respectively (Table 4). The linear mixed effect model of the number of papilloma showed a significant interaction between the time factor (weeks) and the dosage 50 mg and 200 mg. The coefficients of the interaction term between the week and dosage is found to be −0.39 and −0.398 for the dosage 50 mg and 200 mg respectively with values of 0.0049 and 0.0041 respectively. This clearly indicates that the 50 mg and 200 mg treatment dosages have a significant effect on decreasing the number of papilloma. However, since both coefficients are almost identical a 50 mg/kg dosage seems to be optimal.

**Fig. 8:**
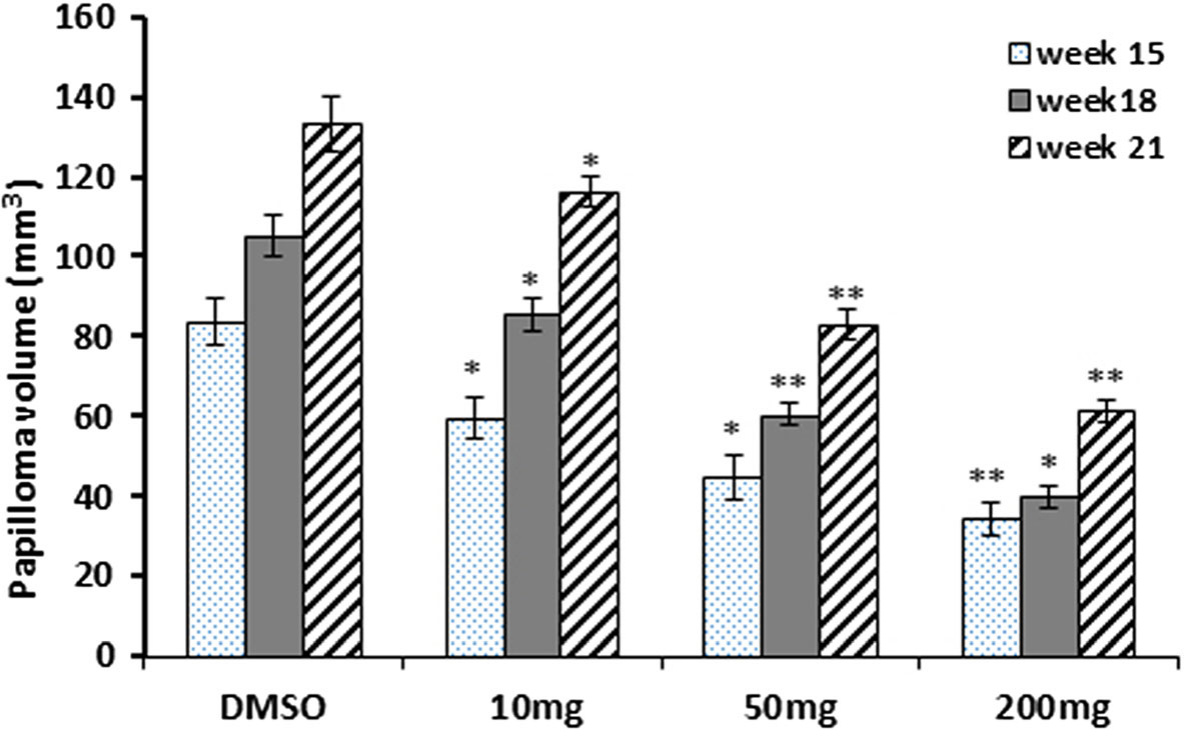
Effect of F2 treatment on the papilloma volume in tumor-bearing mice. Average variations in papilloma volume (mm^3^) of all groups (10, 50 and 200 mg/kg) was measured at weeks 15, 18 and 21 in tumor bearing-mice. Bars represent mean papilloma volume ± SEM. * denotes *P* < 0.05 and ** denotes *P* < 0.01 compared with control (DMSO) mice as measured by one-way ANOVA

**Fig. 9:**
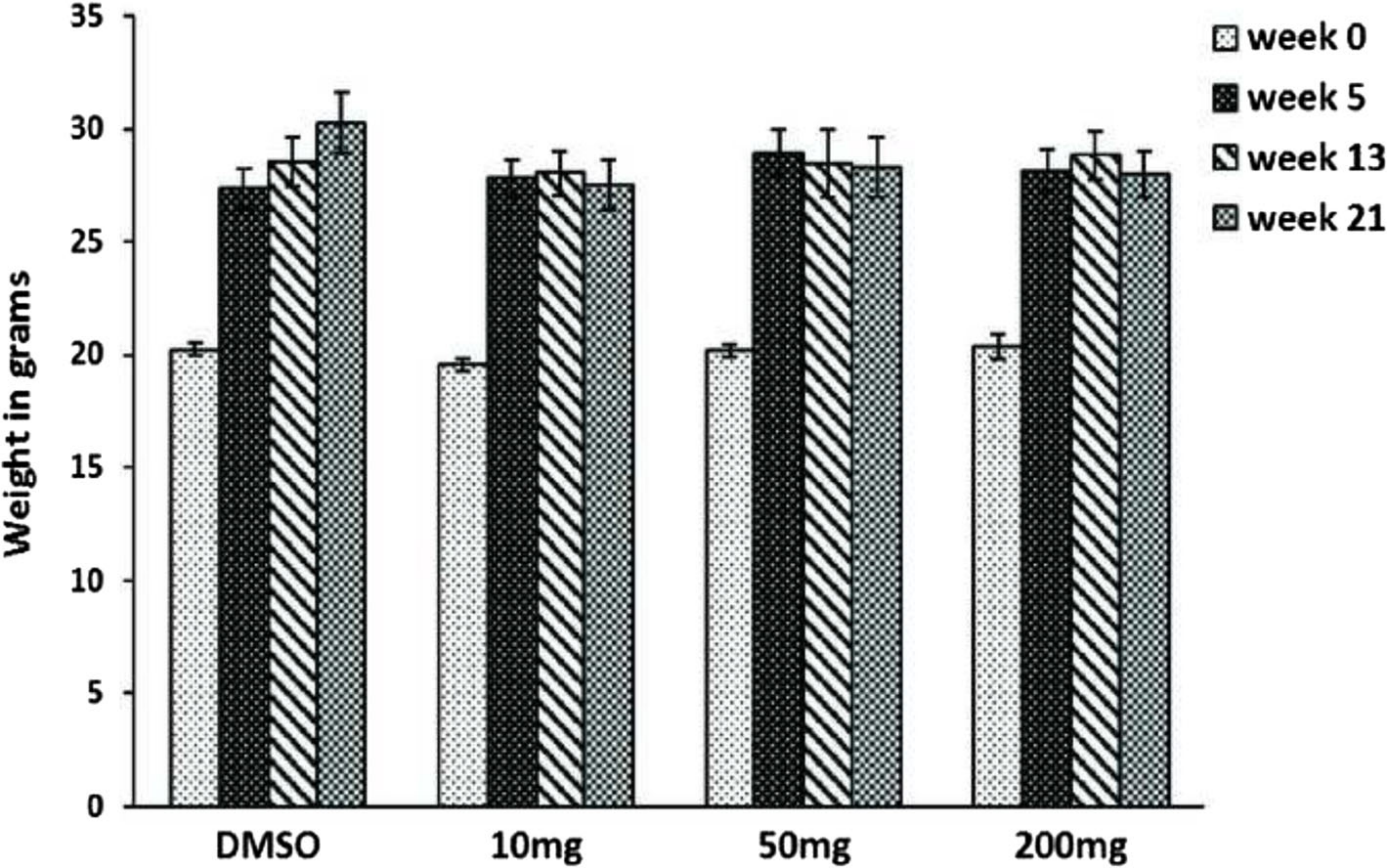
Effect of F2 treatment on the body weight of mice. Average variations in the body weight of the mice of all groups (DMSO, 10, 50, 200 mg/Kg) measured at weeks 5, 13 and 21. Bars represent mean of body weight ± SEM from groups of 10 mice

The effect of the F2 fraction on the volume of the papillomas was also studied (Fig. 8). The linear mixed effect model of the papilloma volume also showed a significant interaction effect between the time factor and the treatment dosage. All treatment dosages (10, 50 and 200 mg/kg) were found to significantly lower the papilloma volume at weeks 15, 18 and 21with *p*-values < 0.05. As shown in Table 5, the total volume of papillomas of control group at week 15 was 83.5 mm^3^. Treatment of animals with F2 resulted in significant inhibition of papilloma volume by 28.5, 46.3 and 58.8%, respectively. Comparable results were obtained at week 18 and at the end of the experiment (week 21) (Table 5). The F2 treated animals did not show significant changes in body weight during the study period compared to the DMSO treated group (Fig. 9).

**Table 5:** The inhibitory effects of different concentrations of F2 fraction on the volume of papilloma (mm^3^) at weeks 15, 18 and 21 in tumor-bearing mice

	Week 15		Week 18		Week 21	
Treatments	Tumor Volume	% Inhibition	Tumor Volume	% Inhibition	Tumor Volume	% Inhibition
Control (DMSO)	83.5 ± 6.0	0	105.3 ± 5.0	0	133.3 ± 6.8	0
10 mg/Kg	59.7 ± 5.3^*^	28.5	85.5 ± 4.2^*^	18.8	116.0 ± 3.6^*^	13.0
50 mg/Kg	44.8 ± 5.7^**^	46.3	60.6 ± 2.8^**^	42.5	82.9 ± 3.8^**^	37.8
200 mg/Kg	34.4 ± 4.2^**^	58.8	40.0 ± 2.7^**^	62.0	61.2 ± 3.0^**^	54.0

Values are mean ± SEM. *n* = 10

^*^ *P* < 0.05, ^**^ *P* < 0.001 vs control

## Discussion

DCOE have been proven lately to possess promising chemopreventive activity against DMBA/TPA-induced skin carcinogenesis [4]. The present study was carried out to evaluate the effects of DCOE fractions in vitro on different tumorigenic HaCaT cells. Additionally, the fraction (F2) containing the major compound 2-himachalen-6-ol (61.4%) was tested in vivo by using the DMBA/TPA chemical carcinogenesis model. Treatment with the different DCOE fractions caused a significant dose-dependent decrease in viability in all treated cells, with F1 and F2 fractions being the most potent. Interestingly, the obtained results revealed a cytotoxic selectivity of the fractions (2.4 – 3 folds) against the tumorigenic variants, as compared to the non-tumorigenic HaCaT cells. Similar to previous studies with human breast [4, 20] and colon cancer (HT-29 Caco-2) cells [14], the F1 and F2 fractions were found to be the most potent ones. The major sesquiterpenes present in F1, the α-humulene and β-caryophyllene, have been reported to be active against various types of human cancer cells [22–24]. On the other hand, the cytotoxicity of the F2 fraction could be primarily due to the presence of the major sesquiterpene 2-himachalene-6-ol which constitutes 61.4%. Flow cytometry analysis on HaCaT-ras II4 and HaCaT-ras A5 cells clearly indicated that F2 treatment induced accumulation of cells in the sub-G1 apoptotic phase and decreases the fraction of cells being in S and G2/M phases. Together this suggests induction of apoptosis and cell cycle arrest by F2. Similar results were obtained on human colon and breast cancer cells after treatment with both F1 and F2 fractions [14, 20]. To explore the possible mechanisms leading to apoptosis induced by F2 treatment, several components of the apoptotic pathways were examined. The present data reveal that treatment of HaCaT-ras II4 cells with F2 caused an up-regulation of the pro-apoptotic Bax and a decrease in the anti-apoptotic protein Bcl-2, resulting in a lower Bcl2/Bax ratio which favors apoptotic cell death. The anti-apoptotic protein, Bcl-2, has been shown to form a complex with Bax protein, thereby preventing its pro-apoptotic activity [25]. Bcl2 protein is known to inhibit apoptosis by preventing Bax translocation to mitochondrial membrane and subsequent release of cytochrome c [26, 27]. The present results also showed that treatment of HaCaT-ras II4 cells with F2 fraction induced a decrease in the level of the inactive caspase-3 indicating its potential cleavage into the activated form. Once activated, the executioner caspase-3 causes the proteolytic cleavage and activation of a wide variety of downstream protein targets [28]. Accordingly, these results suggest that the induction of apoptosis might be mediated via the mitochondrial pathway.

The induction of cell apoptosis by p53 and p21 (WAF1, Cip-1) is well documented [29]. Although p21 is a direct p53 target, it is also known to be regulated by several other tumor suppressors [30]. The current results show that F2 causes an increase in the expression of p21 with a decrease in p53 levels in HaCaT-ras II4 cells. Such observation suggests that the cell cycle arrest and apoptosis in HaCaT-ras II4 cells is mediated through the up-regulation of the expression of p21 in a p53-independent manner. The altered response in p53 expression may be attributed to the presence of mutant p53 in these cells since previous studies conducted in our laboratory have shown that F2 fraction caused p53 up-regulation in HT-29 colon cancer cells [14]. The present findings are in accordance with previous reports indicating that polyphenols such as curcumin [31], and other wine polyphenols including caffeic acid, resveratrol and quercetin [32] down-regulate p53 in cancer cells harboring mutant p53 gene.

Cellular signaling pathways such as the MAPK pathway and PI3K pathway which are involved in controlling cell cycle, cell proliferation and apoptosis are routinely found to be upregulated in cancer cells [33, 34]. ERK1/2 (MAPK) is a transcription factor that leads to the expression of several gene products which positively regulate cell survival [35]. It was reported that constitutively activated ERK is responsible for the increased cell growth in various cancers [35, 36]. Furthermore, activation of ERK 1/2 has also been shown to prevent apoptosis in response to various types of stimuli such as tumor necrosis factor and radiation [37]. Whereas, down-regulation of ERK activity is known to be associated with apoptosis [38]. In the current study, treatment of HaCaT-ras II4 cells with F2 fraction significantly inhibited the phosphorylation of ERK. This down regulation of activation was associated with a decrease in cell survival and proliferation. We speculate that the induction of apoptosis by F2 may be mediated partly through the inhibition of the MAPK/ERK pathway. The AKT protein, main downstream effector of PI3K, is a key mediator of cell survival [39]. AKT activity has been reported previously to be increased in various cancers, including melanoma, colon, brain, breast and prostate [33, 40, 41]. Additionally, the PI3K/AKT survival pathway inhibits apoptosis and promotes tumor cell survival through different types of stimuli such as growth factor withdrawal and loss of cell adhesion [42]. The present data revealed that F2 fraction caused a considerable reduction in AKT phosphorylation in the HaCaT-ras II4 cells, reflecting an inhibition of the PI3K/AKT pathway.

The chemopreventive effect of the F2 fraction was evaluated in DMBA/TPA-induced mouse skin carcinogenesis. The results demonstrate that intraperitoneal injection of F2 fraction at the promotion stage caused a significant reduction in papilloma incidence, yield and volume. These results are consistent with previous work conducted in our laboratory using unfractionated DCOE [4]. In addition, F2 fraction was shown to markedly increase the level of glutathione S-transferase (GST) in CCl4-treated mice [17], an enzyme involved in the detoxification of carcinogens and in turn protect cellular components against toxic compounds and chemical carcinogenesis [43]. Therefore, the chemopreventive action of F2 against DMBA/TPA-induced skin carcinogenesis may be partly attributed to this enhanced activity of GST. Earlier studies in our labs have shown that DCOE percentage yield was 3.47% and contained a major compound constituting around 30% of the crude oil content [4]. In the present study, F2 fraction was shown to be the most active of DCOE fractions and contained around 60% of that major compound. Assuming that it is the major biologically active compound of DCOE, then a 70 kg adult consuming 67 g of the edible umbel would achieve the dose of 10 mg/kg body weight of this compound. The consumption of the whole umbel would provide the consumer with additional anticancer compounds present in F1 fraction and to a lesser extent in F3 and F4 fractions. Since F1 and F2 fraction are the most potent fractions and not water soluble, it is recommended for maximal benefit to consume the whole umbel rather than by infusion form. In the present study, the animals were treated for 21 weeks with doses of 10–200 mg/kg body weight without incidence of death, thus indicating high safety of the F2 fraction. In comparison with a commonly used chemotherapeutic drug, cisplatin, the lowest dose used in the current study is about three times higher. Knowing that it is still a fraction, chromatographic purification of the F2 would most likely lead to the use of a lower and comparable dose to cisplatin.

## Conclusions

The current study demonstrates that *Daucus carota* pentane-diethyl ether fraction (F2) displays an enhanced selective cytotoxicity against the tumorigenic HaCaT-ras variants as compared to non-tumorigenic human HaCaT skin keratinocytes by inducing caspase-dependent apoptotic cell death. The F2-induced cytotoxicity is mainly mediated through inhibition of the MAPK/ERK and PI3K/AKT pathways. The anticancer activity of F2 was also confirmed in vivo using the DMBA/TPA skin carcinogenesis model. Further studies will now be conducted to isolate, chemically characterize and pharmacologically evaluate the main constituents of this F2 fraction.

## Additional files

Additional file 1: Shebaby et al. Raw Data R4. (XLSX 56 kb)
Additional file 2: DCOE Fractions Supplementary Tables and Figures R4. (DOCX 145 kb)
