# Erratum to: Wild carrot pentane-based fractions suppress proliferation of human HaCaT keratinocytes and protect against chemically-induced skin cancer

**DOI:** 10.1186/s12906-017-1661-z

**Published:** 2017-03-09

**Authors:** Wassim N. Shebaby, Mohamad A. Mroueh, Petra Boukamp, Robin I. Taleb, Kikki Bodman-Smith, Mirvat El-Sibai, Costantine F. Daher

**Affiliations:** 1 Department of Natural Sciences, School of Arts and Sciences, Lebanese American University, Byblos, Lebanon; 2 School of Pharmacy, Department of Pharmaceutical Sciences, Lebanese American University, Byblos, Lebanon; 3 Deutsches Krebsforschungszentrum DKFZ, German Cancer Research Center, Genetics of Skin Carcinogenesis, Heidelberg, Germany; 4 IUF–Leibniz Research Institute for Environmental Medicine, Duesseldorf, Germany; 5 Faculty of Health and Medical Sciences, Department of Microbial and Cellular Sciences, University of Surrey, Guildford, UK

## Erratum

Upon publication of the original article [1], it was noticed that the author’s name “Robin I Taleb” was incorrectly given as “Robin I Taleb RI”. This has now also been corrected in the original article.
