# Supplementary material for: Wild carrot pentane-based fractions suppress proliferation of human HaCaT keratinocytes and protect against chemically-induced skin cancer
Source: BMC Complement Altern Med. 2017 Jan 10;17:36. doi: 10.1186/s12906-016-1531-0 (PMC5223476; doi:10.1186/s12906-016-1531-0)
Supplement: Additional file 2: — DCOE Fractions Supplementary Tables and Figures R4. (DOCX 145 kb) [file 12906_2016_1531_MOESM2_ESM.docx]

| Main identified components (> 0.5 %) in the wild carrot oil fractions F1, F2, F3 and F4. | | | | | |
| --- | --- | --- | --- | --- | --- |
| **Retention Time** | **Component** | **Percentage in fractions** | | | |
|  |  | **F1** | **F2** | **F3** | **F4** |
| 11.88 | (+)-α-longipinene | 7.18 | - | 5.60 | 8.18 |
| 12.38 | Longicyclene | 1.72 | - | - | - |
| 13.26 | methyl eugenol | - | 2.28 | - | - |
| 13.33 | 10s,11a-Himachala-3(12),4-diene | 6 | - | - | 0.59 |
| 13.88 | β-Caryophyllene | 17.2 | - | - | 0.78 |
| 15.08 | 1,1,4,8-tetramethyl-cis,cis,4,7,10-cycloundecatriene | - | - | - | 1.25 |
| 15.14 | α-humulene | 28.2 | - | - | - |
| 16.03 | Aromadendrene | 0.75 | - | - | - |
| 16.54 | β-Selinene | 2.86 | - | 2.38 | 5.33 |
| 16.70 | ϒ-Selinene | - | - | 0.56 | 1.86 |
| 16.87 | α-Selinene | 4.26 | - | - | - |
| 16.95 | β-Himachalene | - | 2.07 |  |  |
| 17.01 | (E)- Methylisoeugenol | - | 12.5 | - | - |
| 17.22 | β-Bisabolene | - | - | 0.62 | 2.59 |
| 17.47 | 2,3-Dicyano-6-ethyl-5,7-dimethyl-6H-1,4-diazepine | - | - | - | 3.82 |
| 17.63 | γ-Selinene | 3.53 | - | - | - |
| 17.87 | β-Himachalene | 2.58 | - | - | - |
| 18.31 | 5-Benzyl-4-isopropyl-1H-imidazole | 0.79 | - | - | - |
| 18.38 | (-)-.alpha.-Panasinsen | 1.08 | - | - | - |
| 19.55 | Elemicine | - | 10.9 | - | - |
| 20.50 | Caryophyllene oxide | 5.20 | 0.66 | - | - |
| 23.53 | 2-Himachalen-6-ol | - | 61.4 | 0.79 | 1.67 |
| 25.22 | β-Asarone | - | 1.05 | - | - |
| 28.39 | 3,5,6,7,8,8α-Hexahydro-4,8α-dimethyl-6-(1-methylethenyl)-2(1H)-naphthalenone | 1.92 | - | - | - |
| 61.05 | Methanesulfonic acid, 9-oxabicyclo [3.3.1]non-3-ylmethyl ester | - | - | - | 0.78 |

**MS spectrum of 2-himachalene-6-ol**

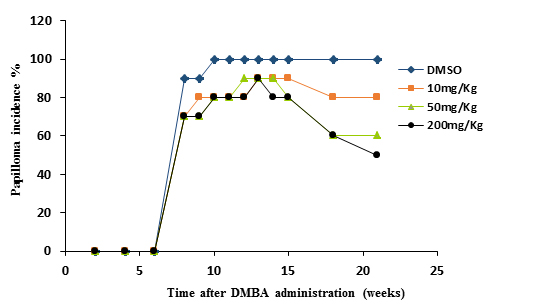


**Figure A.** **Effect of F2 treatment on the papilloma incidence.** Intraperitoneal injections of the F2-fraction (three doses: 10, 50, and 200mg/kg) were performed 30 minutes prior to the application of TPA. The number of mice with papillomas was recorded weekly. Data are expressed as the percentage of mice-bearing papillomas with time (weeks). Each group included 10 mice.

**
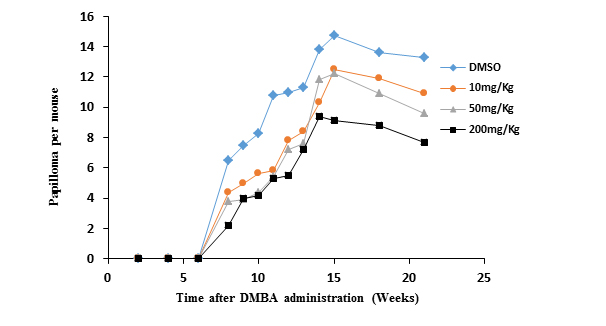
**

**Figure B. Effect of F2 treatment on the number of papilloma per mouse.** Intraperitoneal injections of the F2-fraction (three doses 10, 50, and 200mg/kg) were performed 30 minutes prior to the application of TPA. The number of papillomas per mouse was recorded weekly. Each group included 10 mice.
